# A comparison of patient appraisal of professional skills for GPs in training participating in differing education programs

**DOI:** 10.1186/s12909-022-03733-9

**Published:** 2022-09-10

**Authors:** Ajit Narayanan, Caitlin Vayro, Michael Greco, Dale Hanson, Jan Hanson, Neil Spike, Pat Giddings, Ben Mitchell, Rebecca Stewart

**Affiliations:** 1 School of Engineering, Computer and Mathematical Sciences, Auckland University of Technology, Auckland, New Zealand; 2 General Practice Training Queensland, Brisbane, QLD Australia; 3 School of Medicine and Dentistry, Griffith University, Brisbane, QLD Australia; 4 Client Focused Evaluation Programme (CFEP Surveys), Everton Park, Brisbane, QLD Australia; 5 College of Public Health, Medicine and Veterinary Sciences, James Cook University, Townsville, QLD Australia; 6 Practice Experience Program, General Practice Training Queensland, Brisbane, QLD Australia; 7 Eastern Victoria General Practice Training, Hawthorn, VIC Australia; 8 Remote Vocational Training Scheme Ltd, Albury, NSW Australia; 9 General Practice Clinical Unit, Faculty of Medicine, The University of Queensland, Brisbane, QLD Australia; 10 Education Services, Training Programs, Royal Australian College of General Practitioners, East Melbourne, VIC Australia

**Keywords:** Patient assessment, GPs in training, Professional development, Multisource feedback, International medical graduates, Patient reported experience measure (PREM)

## Abstract

**Background:**

Medical boards and healthcare providers internationally are coming under increasing pressure to attract international medical graduates (IMGs) and overseas trained doctors (OTDs) to cope with predicted general practice (GP) doctor shortages. Various pathways to registration are made available for this purpose. There is very little understanding of the effects of different training pathways to licensing and registration on the ability of IMGs and OTDs, as well as locally trained doctors, to acquire the desirable professional skills deemed necessary for working effectively in the primary care sector.

**Methods:**

Feedback from patients was collected at the end of their scheduled consultation with their doctor using a questionnaire consisting of 13 Likert scale items that asked them to rate their experience of the consultation. Feedback was obtained for doctors going through the Royal Australian College of General Practice (RACGP) Practice Experience Program (PEP) and the Australian General Practice Training Program (AGPT), with the former intended primarily for IMGs and OTDs, and the latter for local medical graduates including from New Zealand. Patient feedback was also obtained for patients visiting already Fellowed and experienced GPs for comparative purposes, resulting in data for three groups of doctors (two trainee, one already Fellowed). Rater consistency and agreement measures, analysis of variance, principal component analysis, t-tests and psychometric network analysis were undertaken between and within groups to identify similarities and differences in patient experience and professionalism of doctors.

**Results:**

There was a small but significant difference in average patient raw scores given to PEP and AGPT doctors (90.25, 90.97%), with the highest scores for ‘Respect shown’ (92.24, 93.15%) and the lowest for ‘Reassurance’ 89.38, 89.84%). Male patients gave lower scores (89.56%) than female patients (91.23%) for both groups of doctors. In comparison, patients gave experienced GPs an average 91.38% score, with male patients giving a lower average score than female patients (90.62, 91.93%). Two components were found in the patient data (interpersonal communication, caring/empathy) that account for over 80% of the variance. When patient scores were aggregated by doctor, the average PEP and AGPT doctor scores received were 90.27 and 90.99%, in comparison to the average experienced GP score of 91.43%. Network analysis revealed differences in the connectedness of items between these two groups as well as in comparison with experienced GPs, suggesting that PEP doctors’ skills are less cohesively developed in the areas of listening ability, explaining and providing reassurance.

**Conclusions:**

The small but statistically significant differences between doctor groups reported in this preliminary study are supplemented by percentile analysis, network analysis and principal component analysis to identify areas for further exploration and study. There is scope for improving the integration of interpersonal communication skills of GPs in Training with their caring and empathy skills, when compared with experienced GPs as a benchmark. Suggestions are made for enhancing professional skills from a patients’ perspective in future training programs.

**Supplementary Information:**

The online version contains supplementary material available at 10.1186/s12909-022-03733-9.

## Background

Healthcare systems internationally are under increasing pressure to recruit and retain physicians given worldwide demand that exceeds supply. The World Health Organization noted in 2006 that shortage of physicians was likely to be widespread in many countries by 2015 [1]. Current predictions are that the USA, for example, will have a shortage of between 21,000 to 55,000 primary care physicians by 2033 [2]. Also, there are predictions that there will be a shortage of over 9000 GPs by 2030 in Australia, representing almost a quarter of the workforce [3]. Clearly, there is a need for suitably qualified GPs, adequately skilled to provide professional and empathic care in the context of the healthcare system in which they work.

Internationally, there is increasing awareness that policies and strategies for increasing the number of international medical graduates (IMGs) and overseas trained doctors (OTDs, a term used to describe doctors who obtained their primary medical qualification in a country apart from Australia and New Zealand) in overburdened healthcare systems need to focus on enhancing pathways to allow such doctors to be registered and credentialed, so that they can practice effectively in their newly adopted country. Three policy issues under current debate include historical bias in the registration process, making it more difficult for IMGs and OTDs to qualify than locally trained doctors [4, 5]; increased risk of complaints against IMGs and OTDs [6] should pathways be eased; and racism and bias against IMGs and OTDs at both systemic and individual levels [7]. There is also a perception that increased risk of complaints may be due not to lack of clinical skills but of professional or ‘soft skills’, such as interpersonal communication skills and empathy, where different cultural backgrounds can lead to different use of language and interactions with patients [5].

Given the reliance on IMGs and OTDs for dealing with growing shortfalls in primary care, there needs to be more understanding of how the professional performance of IMGs and OTDs compares with their locally trained counterparts. Such understanding may identify improvements in aspects of IMG and OTD training as well as help these doctors to better understand the needs and expectations of their intended national healthcare system and its patients. Previous comparative studies on professional performance of IMGs and OTDs have tended to use outcomes such as patient survival [8] and complaint rates [6], or simulated case studies [7]. These outcomes, while important, do not focus on the skills that contribute to professional performance.

In Australia, 29% of the current medical workforce consists of doctors who have trained overseas [9], and many of these doctors also identify difficulties that relate to their performance. For example, IMGs and OTDs have reported struggling when attempting the Australian Medical Council Examinations [10]. There is also literature exploring the reasons doctors who have trained overseas may have difficulty working in Australia and it has been identified that the process of migration and adjustment has affected their performance [11]. Further, a difference in personality traits of internationally trained doctors compared with Australian graduates has been demonstrated and may provide some insight into their professional attributes, and performance [12]. Other factors identified have included difficulty with English, differences in medical education, length of time since medical school graduation, family and financial obligations, cultural approaches and beliefs, and the status and role of the physician [13]. While these studies help build holistic understanding into the professional performance of IMGs and OTDs, and highlight some important factors to consider, they do not directly investigate the skills of these doctors transitioning into the Australian medical system.

There has been very little attempt to identify the effect of different General Practice education and training programs on the ability of IMGs and OTDs, as well as locally trained doctors, to acquire the desirable professional skills deemed necessary for working effectively in the general practice sector. In particular, there appears to be no detailed study of how doctors gaining General Practice specialist registration through the different programs are perceived by patients in terms of their professional skills. Finally, there appears to have been no comparison between patient perception of General Practitioners in Training (GPiT) on the one hand, and patient perception of experienced practitioners on the other, to identify possible areas for enhancement of professional skills for both types of fellowship programs.

This study seeks to understand the professional performance of doctors, as perceived by patients, with a particular focus on doctors who have gained their primary qualification overseas. Previous cultural factors can be expected to influence how this group communicates with patients [11, 14]. Many doctors undertaking the Royal Australian College of General Practice (RACGP) Practice Experience Program (PEP) obtained their primary qualifications overseas and are working in areas of workforce shortage under limited or no formal supervision [15]. PEP is a self-directed education program delivered in partnership with training organisations to support doctors gain RACGP Fellowship, thereby allowing them to continue to practice in Australia in the primary care sector as GP specialists. The Australian General Practice Training (AGPT) program, on the other hand, prepares mainly Australian and New Zealand medical graduates for RACGP Fellowship and specialist registration by providing a three- or four-year educator-directed training program, including intensive supervision. Eligibility for the AGPT is more restricted than for the PEP with a subsequent competitive selection process. AGPT program training takes place in hospitals and general practices. The AGPT program currently is the most common pathway for Australian registrars to achieve General Practice Fellowship. Doctors on both programs are GPs in Training (GPiTs). Further details of the two programs and their differences using the TIDieR checklist as a guide [16] can be found in Table 1, with the latest information on the demographics of doctors involved available via the RACGP website [17].

**Table 1:** Overview of AGPT and PEP training programs using the TIDieR checklist as a guide

**Intervention: A comparison of patient appraisal of professional skills for GPs in Training participating in differing education programs.**		
**Rationale:** To understand the performance of doctors on differing pathways to licensing and registration as specialist GPs in the desirable professional skills deemed necessary for working effectively in the primary care sector.		
**Materials and Procedures:** Both groups received the Multisource Feedback tool which comprised patient and colleague feedback, and self-reflection. These surveys were administered as a requirement for education and training in Australian General Practice.		
**Comparison of education programs**		
**Descriptor**	**Australian General Practice Training program**	**Practice Experience Program**
Participant demographic	31% over the age of 35 Majority female (60%) [17]	86% over the age of 35 Majority male (56%)
Country of primary medical qualification	Australia	Outside of Australia
Program length	3–4 years full-time	12–36 months part or full-time
Education and Training	Education and training program including uniformly mandated supervision and progress monitoring including assessment of learning.	Self-directed education program with variable supervisory requirements.
Practice location	Major cities (50%) [17]	Predominantly outside of major cities (78%)
Practice scope	Working in comprehensive General Practice settings with diversity of practice scope.	Working in varied General Practice settings, including those with limited practice scope.
Curriculum	Formal syllabus-based individual and small group teaching based on the RACGP Curriculum.	Self-directed learning based on the RACGP Curriculum.
Assessment	Workplace Based Assessment for the determination of progress with mandated remediation where required. i.e. assessment of learning.	Workplace Based Assessment for the purposes of feedback. i.e. assessment for learning
MSF activity	MSF not mandatory	MSF mandatory
	**End point:** Summative written and oral RACGP examinations contributing to eligibility for Fellowship of the RACGP.	

The overall aim of our study is to compare patients’ experiences of the professionalism of GPiTs on the two different training programs given their distinctly different cohort demographics. Understanding any differences can lead to improvements in training programs, peer-dialogue, and reflection for the benefit of patients. To provide a benchmark against which both program groups can be measured, a third and large dataset of patient ratings for current (Fellowed) GPs undertaking patient feedback as part of their continuing professional development (CPD) for ongoing Medical Board of Australia registration was used. Since this third group will already have had several years’ experience in the primary care sector and the majority have achieved GP specialist registration, their professional performance as rated by patients can provide standards and a benchmark to which the two groups of trainee practitioners may wish to aspire. Patient data from these three groups are labelled Dataset A, Dataset B and Dataset C below.

## Methods

### Overview

Patient feedback was obtained for doctors going through the Royal Australian College of General Practice (RACGP) Practice Experience Program (PEP) and the Australian General Practice Training Program (AGPT), with the former intended primarily for IMGs and OTDs, and the latter for local medical graduates including from New Zealand. Further details of the two programs and their differences using the TIDieR checklist as a guide [16] can be found in Table 1: Comparison of education programs. This study deals with the patient feedback aspect of the two progams only. Patient feedback was also obtained for patients visiting experienced GPs for comparative purposes, resulting in data for three groups of doctors (two trainee, one experienced).

### Data

The data consist of 57,745 anonymized patient responses to three groups of doctor (average 36–39 patients per doctor type) working in Australia, resulting in three datasets:

- Dataset A. 221 doctors who have trained primarily overseas and who are enrolled in the RACGP PEP;
- Dataset B. 355 General Practice registrars enrolled in the AGPT Program; and
- Dataset C. 923 Australian GPs who receive patient feedback as part of their CPD program (GP CPD).

The patient questionnaire used in this study deals with the patient’s visit to their doctor and asks patients to rate their just completed consultation experience. PEP and AGPT patient questionnaires use 13 questions, and GP patient questionnaires use 12 of the 13 questions (more details below). All questions ask for responses using a five-point Likert scale with labels ‘poor’, ‘fair’, ‘good’, ‘very good’, and ‘excellent’. Additional file 1 provides the full text of each question, with a shortened version as used in this report.

### Data collection

A Human Research Ethics Committee approved this study (clearance number 2020000515 from the University of Queensland). The participants gave informed consent for their non-identifiable data to be used in research as part of the consent process to undertake feedback. The data were collected in the period between 1st January 2018 and 30th April 2020. A pack of 50 questionnaires per participating doctor was sent to practices, with written instructions provided to practice reception staff to hand out the questionnaire to consecutive patients so that convenience sampling based on willingness to participate was implemented. Patients were asked to rate their encounter according to their experience of that specific visit. To ensure patient confidentiality and anonymity, and to encourage honest feedback, completed questionnaires were placed in self-sealed envelopes and into ballot-style boxes by patients themselves before departure from the practice. No post-departure completion through email or internet took place.

Further details concerning the content and format of patient questionnaire can be obtained by emailing the authors.

Questionnaires were processed by Client Focused Evaluation Program (CFEP) Surveys in Brisbane, Australia. Paper questionnaires were scanned and verified electronically by an experienced data auditor. Data were imported to an in-house software system running on an enterprise database where they were further checked and verified. The patient datasets were exported as Microsoft Excel Spreadsheets to an SPSS database (SPSS for Windows Version 25) and cleaned and checked prior to data analysis.

### Statistical analysis

On the basis that the intervals between the five Likert scale points are equal, item responses were converted into percentages (‘poor’ = 20%, ‘fair’ = 40%, ‘good’ = 60%, ‘very good’ = 80%, ‘excellent’ = 100%) to allow for parametric techniques based on means, standard deviations and variances. Conversion to percentages can aid intelligibility, allow benchmark comparison across different studies and groups as well as highlight differences without the need to represent results to four or five decimal places. Presenting percentages also provides consistency with previous doctor feedback results in the Australian professional performance framework that have been presented in percentages [18]. Two levels of analysis were conducted: at the raw score rater- and item- level (irrespective of doctor rated), and at the aggregated doctor level where doctors received the mean item scores of all their raters.

The sampling strategy detailed above has special characteristics that need to be accounted for, that is, the data are unbalanced because of variable numbers of raters per ratee, fully nested because all the ratees may be unique to that rater, and uncrossed because raters provide only one rating per ratee on one occasion. Cronbach’s alpha is reported below, but the alpha results should be interpreted with caution since some of the assumptions of its use (e.g., all raters are rating the same subject, object, or event) are not met in this study. Its use here is to check on the internal consistency of the questionnaire (questionnaire reliability). A signal-to-noise ratio (SNR) measure for dealing with unbalanced, uncrossed and fully nested data is also used to provide an estimate of data reliability [18]. The content and construct validity of the original patient questionnaire were first established in 1999 [19] as the Doctor’s Interpersonal Skill Questionnaire (DISQ) and its validity re-evaluated in 2010 when being assessed for use in the relicensure of doctors by the UK GMC [20]. Its validity and reliability were reaffirmed in 2013 after minor edits were made to the wording of some items and the revised questionnaire applied in unbalanced, uncrossed and fully nested studies involving over 85,000 patients to over 2000 doctors [21].

Analysis of variance (ANOVA) is a collection of statistical models used to analyze the differences among group means. The observed variance in a particular variable is partitioned into components attributable to different sources of variation. In its simplest form, ANOVA provides a statistical test of whether or not the means of several groups are equal. ANOVA is used to test for differences in item ratings and averages within and between PEP, AGPT and GP CPD data.

Principal component analysis (PCA) is a data reduction technique for explaining variance in data using a smaller set of variables than the original variables or items. The Kaiser-Meyer-Olkin (KMO) test is a measure to determine sampling adequacy for each item. KMO values between 0.8 and 1.0 indicate that there are enough samples and sufficiently low variance for efficient identification of underlying components. Bartlett’s sphericity tests whether there are relations among variables suitable for structure detection, and PCA is used here to confirm the presence of components previously found when demonstrating criterion and construct validity [18]. Single measures intraclass coefficients (ICCs) provide a relative measure of the variability in the sample of responses and is useful for estimating the agreement between raters on how to interpret the items. Values between 0.4 to 0.6 are considered ‘moderate agreement’, between 0.6 and 0.8 ‘good agreement’ and above 0.8 ‘very good agreement’ [22]. One-way random ICCs are used in this study to check for reliability of the questionnaires given that all the raters are different.

A t-test compares the means of two variables to determine whether there is a difference. Such a test can be used to estimate whether the responses given by two populations to a single set of items differ significantly. T-tests assume a normal distribution and the raw score data in this study are negatively skewed. However, its use is justified here because of the large sample sizes and the need to check whether item means differ between groups of doctors after aggregation by doctor (distribution of distributions). Linear regression is used to estimate and control for possible bias in ratings due to sociodemographic factors. These factors are entered first into the regression model against the dependent variable (average patient score) followed by entry of the independent variables (questionnaire items), with comparisons made concerning the amount of variance explained at each step.

Psychometric network analysis provides graphical representations of relationships and interactions between variables such as questionnaire items [23]. Nodes in the graph represent the items and links represent the strength of association between them. Inter-item mean score correlations, scaled between 0 and 1 are used, with width of links proportional to the strength of the association. The layout adopted is the force-directed ‘spring’, where variables with strongest associations and therefore of hypothesized strongest influence are placed closer together and at the centre of the graph [24]. Summing the correlations for each item results in a node ‘strength’ measure that can be useful for assessing the influence of items and identifying possible points for future intervention, based on the assumption that changes in central items should have greatest impact on other items. Centrality scores are presented as standard scores (standard deviations above or below mean 0) to allow for comparison across the different doctor groups. All statistical analysis was carried out with SPSS v25 and network analysis through qgraph in R.

## Results

Overall, while scores fell in the ‘very good’ to ‘excellent’ range, there were small but statistically significant differences between patient scores for PEP doctors and AGPT registrars at both the raw score and aggregated levels, with PEP doctors scoring lower. GP CPD doctors received the highest scores, especially on items dealing with confidence in their ability and satisfaction with the visit. Patients aged 25 and below gave the lowest scores to all doctor groups. Patients seeing their regular GP gave higher scores than other patients. Internal consistency and reliability of the questionnaires and data were acceptably high. Confirmatory PCA identified the same underlying assessment components as previous, comparable studies. Network analysis revealed that ability to listen was central to patient perceptions of PEP doctors, whereas concern for patient was central to AGPT program doctors. More detailed analysis now follows.

### Patient data (raw scores)

Table 2 provides an overview of the patient data across the three datasets. There were 7907 patient responses to 221 PEP doctors (Dataset A), and 13,623 patient responses for 355 AGPT registrars (Dataset B). The average patient raw score (irrespective of doctor rated) on all 13 items was 90.25% for PEP (SD = 12.92) and 90.98% (SD = 12.08) for AGPT, indicating an overall patient response tending towards the higher end of the ‘very good’ to ‘excellent’ range. Post-hoc power analysis showed 98.1% power for these means, SDs and sample sizes for detecting differences at 0.05 significance level. For both PEP and AGPT patients, the highest scoring item was ‘Respect shown’ (92.24 and 93.15%, respectively), and the lowest ‘Reassurance’ (89.38 and 89.84%, respectively. The average rate of missing responses was very low for both PEP and AGPT patients (0.32, 0.31%), with the highest being for ‘Take care of myself’ (0.9, 0.62%) and the lowest ‘Warmth of greeting’ (0.1, 0.06%).

**Table 2:** Overview of the data across the three datasets

Responses	PEP	AGPT	GP CPD
Number of patients	7907	13,623	36,215
Number of doctors	221	355	923
Number of female patients (%)	4697 (59.4%)	8680 (63.7%)	21,739 (60%)
Number of male patients (%)	3014 (38.1%)	4454 (32.7%)	13,109 (36.2%)
Patients under 25 (%)	1086 (13.7%)	2336 (17.1%)	3010 (8.3%)
Patients 26–59 (%)	4178 (52.8%)	7480 (54.9%)	18,119 (50%)
Patients 60+ (%)	2482 (31.4%)	2482 (24.7%)	14,248 (39.3%)
Usual GP	4835 (61.1%)	3834 (28.1%)	29,000 (80.1%)
Not usual GP	2637 (33.4%)	8747 (64.2%)	5769 (15.9%)
Average patient score (SD)	90.25 (12.92)	90.98 (12.08)	91.39 (12.27)
Average doctor score (SD)	90.27 (6.32)	90.99 (4.87)	91.43 (5.19)
Lowest doctor score	60.69	65.50	64.29
Highest doctor score	99.16	98.13	100.00

**Fig. 1:**
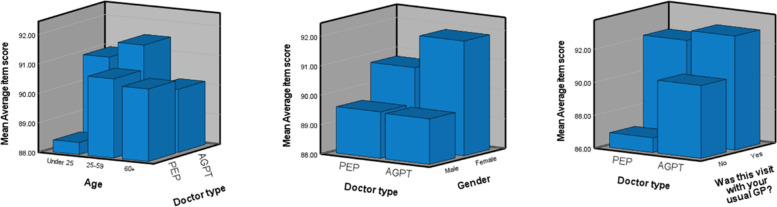
Average raw patient scores (y axis) compared by PEP and AGPT doctor type and patient age (left), gender (middle) and usual visit (right). Note that the y-axis has been constrained to make the differences clearer

Of the PEP patients, 13.7% were under 25 years of age (*n* = 1086), 52.8% between 25 and 59 (*n* = 4178) and 31.4% over 60 (*n* = 2482). The corresponding AGPT patient figures were 17.1% (*n* = 2336), 54.9% (*n* = 7480) and 24.7% (*n* = 2482), respectively (Fig. 1). PEP patients under 25 gave a significantly lower average score (88.4%, *p* ≤ 0.05) than both patients aged 25–59 (90.7%) and patients over 60 (90.5%). AGPT patients between 25 and 59 gave significantly higher average scores (91.53%, *p* ≤ 0.05) than patients under 25 (90.99%) and over 60 (90.14%).

With respect to gender, 59.4% of PEP patients and 63.7% of AGPT patients were female, while 38.1 and 32.7%, respectively were male. There were 2.5 and 3.6% of PEP and AGPT patients, respectively, who did not declare their gender. Male patients from each pathway gave a significantly lower average score (89.58% PEP, 89.54% AGPT) than their corresponding female patients (90.79% PEP, 91.89% AGPT, *p* ≤ 0.01). The majority of PEP patients (61.1%) reported that their visit was with their usual doctor, whereas only 28.1% of AGPT patients reported the same. Both groups of patients who saw their usual doctor gave an average score of just over 92%. However, for patients who reported not seeing their usual doctor, there as a marked difference in the average score, with patients seeing PEP participants giving 86.9% in comparison to 90.4% given by patients consulting AGPT registrars (Fig. 1).

The GP CPD data (Dataset C) consists of 36,215 patient responses to 923 GPs undertaking CPD programs. GP CPD patient questionnaires used the same items as the PEP and AGPT questionnaires with the exception of the item ‘Take care of yourself’. This item was removed since many GPs are typically located in large practice settings where a number of other practice staff (e.g., nurses, practice managers, and physiotherapists) are also involved in this patient aspect. The average patient score on the 12 GP CPD items was 91.39% (SD = 12.27). The highest scoring item was ‘Respect shown’ (92.93%) and the lowest ‘Reassurance’ (90.44%). The average rate of missing responses was 0.9%, with the highest (1.3%) being for ‘Time for visit’ and the lowest (0.4%) for ‘Warmth of greeting’.

Analysis of demographic data showed that 8.3% of GP CPD patients were under 25 years of age (*n* = 3010), 50% between 25 and 59 (*n* = 18,119) and 39.3% over 60 (*n* = 14,248). Similarly to the PEP patients, GP CPD patients under 25 gave a significantly lower average score (89.66%, *p* ≤ 0.01) than patients aged 25–59 (91.57%) and patients over 60 (91.65%). With respect to gender, 60% of GP CPD patients were female, 36.2% were male, and 3.8% did not declare their gender. Male patients gave a significantly lower average score (90.62%, *p* ≤ 0.01) than female patients (91.93%), as was seen with PEP and AGPT patients. The majority of GP CPD patients (80.1%) reported that their visit was with their usual GP, whereas 15.9% reported that it was not. Patients reporting seeing their usual GP gave a significantly higher score (92.26%) than those who did not see their usual GP (87.5%, *p* ≤ 0.01).

One-way random ICC across all 12–13 items for each of the three patient datasets was 0.77, indicating good agreement among the different raters for interpreting the questionnaire items. Additionally, Cronbach’s alpha was a high 0.97, indicating high internal consistency of the questionnaire irrespective of the type of doctor being rated. The average inter-item correlation varied between *r* = 0.76 and *r* = 0.78 for all three datasets. SNR estimates [18] were in the range 0.89 to 0.90 for all three datasets, indicating that 89–90% of the data was likely to be true data and the rest due to noise and error from interactions between raters, items, and ratees.

### Estimating the effect of patient demographics and item removal

For all three doctor groups, patient age and gender contributed less than 0.5% (adjusted R^2^ ≤ 0.005) of the variance in average patient scores. Patients seeing their usual doctor contributed 4% (adjusted *R*^2^ = 0.039) to PEP patient average score, less than 1% (adjusted *R*^2^ = 0.009) to AGPT patient average score, and less than 2% (adjusted *R*^2^ = 0.019) for GP CPD patient average score. The 12–13 Likert items contributed the remaining 96 to 98% of the variance. Aggregation of raw score patient data at the doctor level was undertaken without adjustment for demographic factors (Section 3.4 below). The item ‘Take care of myself’, which is not part of the GP CPD items but is part of the PEP and AGPT items, contributed just 0.1% (adjusted *R*^2^ = 0.001) of the variance to PEP and AGPT patient average score after taking into account the other 12 items. Patient GP CPD average scores are therefore unlikely to be impacted by its absence.

### Principal component analysis of patient data

A Kaiser-Meyer-Olkin (KMO) sampling adequacy measure of 0.98 and a significant Bartlett’s test for sphericity (*p* ≤ 0.001) indicated that PCA was appropriate. Confirmatory PCA using the varimax rotation method (to spread the highly loaded items across the components) revealed two previously identified primary dimensions known to belong to patient-doctor professional relationships, namely, interpersonal communication (component 1) and caring/empathy (component 2) [25], thereby establishing criterion (external) validity (Table 3). ‘Satisfaction with the visit’ (item 1) was related to interpersonal communication in line with previous studies [18], thereby establishing construct validity. The amount of variance explained by the two components was over 80% for each group of doctors.

**Table 3:** Principal component analysis (varimax method of rotation) of raw score patient data showing two components ‘interpersonal communication’ and ‘caring/empathy’ for all three doctor groups. Only the highest loadings are shown

Components	PEP patients		AGPT patients		GP patients	
	1	2	1	2	1	2
Item						
Satisfaction with visit	0.80		0.80		0.81	
Warmth of greeting	0.78		0.78		0.82	
Ability to listen	0.74		0.72		0.76	
Explanations	0.73		0.70		0.70	
Reassurance	0.70		0.71		0.67	
Confidence in ability	0.69		0.70		0.67	
Express concerns		0.68		0.69		0.70
Respect shown		0.68		0.70		0.68
Time for visit		0.82		0.81		0.82
Consideration		0.77		0.76		0.81
Concern for patient		0.74		0.77		0.78
Take care of myself		0.75		0.75		
Recommendation		0.66		0.65		0.69
*Variance explained*	41.02%	40.58%	39.93%	40.81%	42.35%	41.16%

### PEP, AGPT and GP CPD doctors (mean scores)

For PEP doctors there was an average of 35.78 patients per doctor (SD = 4.11, minimum 30, maximum 48, response rate 72%), with a mean PEP doctor score of 90.27 (SD = 6.32, range 60.69–99.16, *n* = 221). The floor effect based on bottom 15th percentile was 85 and the ceiling effect based on top 15th percentile was 95.71. The lower and upper quartiles were 87.98 and 94.16.

For AGPT doctors, there was an average of 38.37 patients per doctor (SD = 8.04, minimum 30, maximum 96, response rate 77%), with a mean AGPT doctor score of 90.99 (SD = 4.87, range 65.6–98.13, *n* = 355). The floor and ceiling effects were 86.67 and 95.26, and lower and upper quartiles were 88.81 and 94.31, respectively.

For GP CPD, the mean score was 91.43 (Average patients per doctor = 39.24, SD = 5.19, range 64.29–100, *n* = 923, response rate 78%), with floor and ceiling effects of 86.77 and 96.3, and lower and upper quartiles of 88.87 and 95.19, respectively. The average score difference between experienced GPs and trainees was 0.79, with the largest individual item differences being in ‘Confidence in ability’ and ‘Satisfaction with visit’ (Table 4).

**Table 4:** Comparison of doctors’ scores from patients, with differences calculated for AGPT doctors scores minus PEP doctor scores, and GP CPD scores minus the average of PEP and AGPT scores

Item	PEP (***n*** = 221)	AGPT (***n*** = 355)	GP CPD (***n*** = 923)	Difference AGPT − PEP	Difference GP CPD − PEP/AGPT
Q1 Satisfaction with visit	89.82	90.06	91.29	0.24	1.35
Q2 Warmth of greeting	90.86	91.46	92.03	0.60	0.87
Q3 Ability to listen	90.74	91.73	91.82	0.99	0.58
Q4 Explanations	89.50	90.63	91.08	1.13	1.02
Q5 Reassurance	89.38	89.84	90.44	0.45	0.83
Q6 Confidence in ability	90.05	89.96	92.04	−0.09	2.03
Q7 Express concerns	89.77	90.81	90.81	1.04	0.52
Q8 Respect shown	92.24	93.16	92.93	0.91	0.23
Q9 Time for visit	89.40	90.99	90.16	1.60	−0.04
Q10 Consideration	90.21	91.04	91.03	0.83	0.41
Q11 Concern for patient	90.56	91.35	91.51	0.78	0.56
Q12 Take care of myself	89.88	90.57		0.69	
Q13 Recommendation	91.11	91.39	91.99	0.29	0.74
Average	*90.27*	*90.99*	*91.43*	*0.72*	*0.80*

**Fig. 2:**
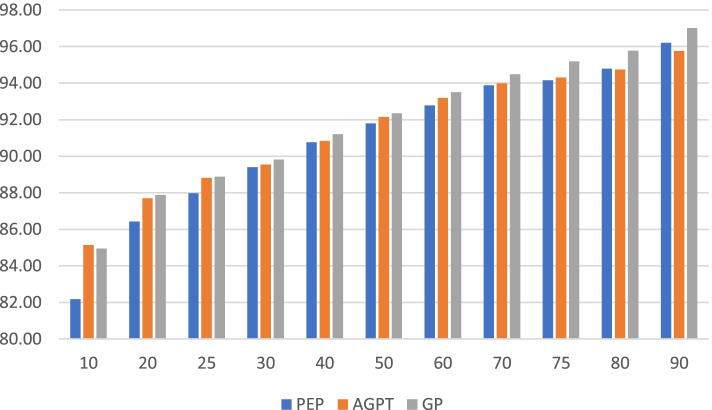
Comparison of PEP (*n* = 221), AGPT (*n* = 355) and GP CPD (*n* = 923) doctors’ mean score received from patients (y axis) by percentile (x-axis). Note that the y axis has been constrained to make the differences clearer

Multiple t-tests showed that PEP doctors received significantly lower item scores than AGPT doctors and GP CPD doctors (*p* ≤ 0.01). While there was a tendency for AGPT doctors to receive lower scores on some items than GP CPD doctors, this was not significant (*p* = 0.13). Comparison by percentiles (Fig. 2) showed that GP CPDs had significantly higher scores than each of the other two doctor groups across all 10 percentiles (*p* ≤ 0.01). There was no significant difference in scores by percentile between PEP and AGPT doctors.

### Network analysis comparisons between doctor groups

**Fig. 3:**
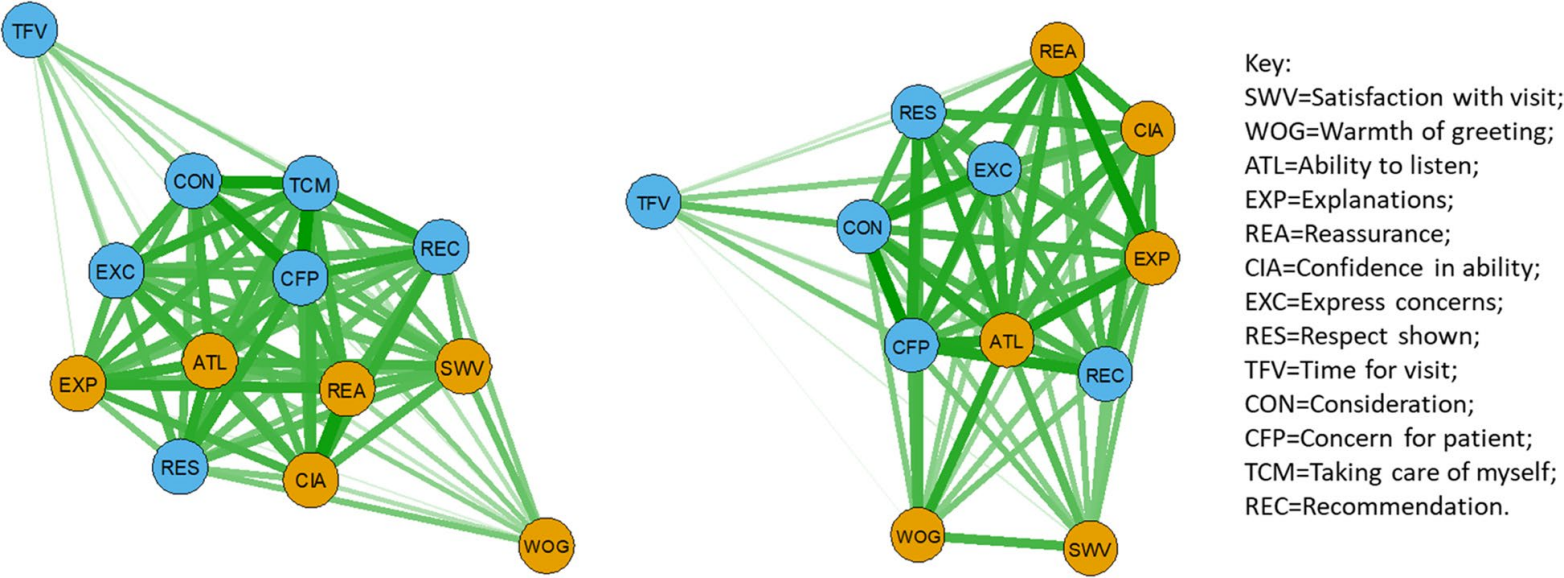
Psychometric network analysis showing mean-score item associations for (left) PEP/AGPT combined doctors (*n* = 576) and (right) GP CPD doctors (*n* = 923), with thickness of line related to strength of association (inter-item correlations rescaled between 0 and 1). The nodes are grouped by principal component (brown = interpersonal communication, blue = caring, Table 3) and the layout is ‘spring’

Psychometric network analysis [23] using correlations between item mean scores revealed that, for PEP and AGPT doctors combined, the central associations were between ‘Concern for patient’, ‘Ability to listen’ and ‘Reassurance provided’ (Fig. 3, left). ‘Respect shown’ was also strongly associated with ‘Ability to listen’. For GP CPD, several other strong associations were apparent (Fig. 3, right). In particular, ‘Ability to listen’ was strongly associated with ‘Explanations’ and ‘Concern for patient’. ‘Time for visit’ lay at the periphery of both networks, and ‘Warmth of greeting’ was more peripheral for PEP/AGPT doctors than for GP CPD doctors.

**Fig. 4:**
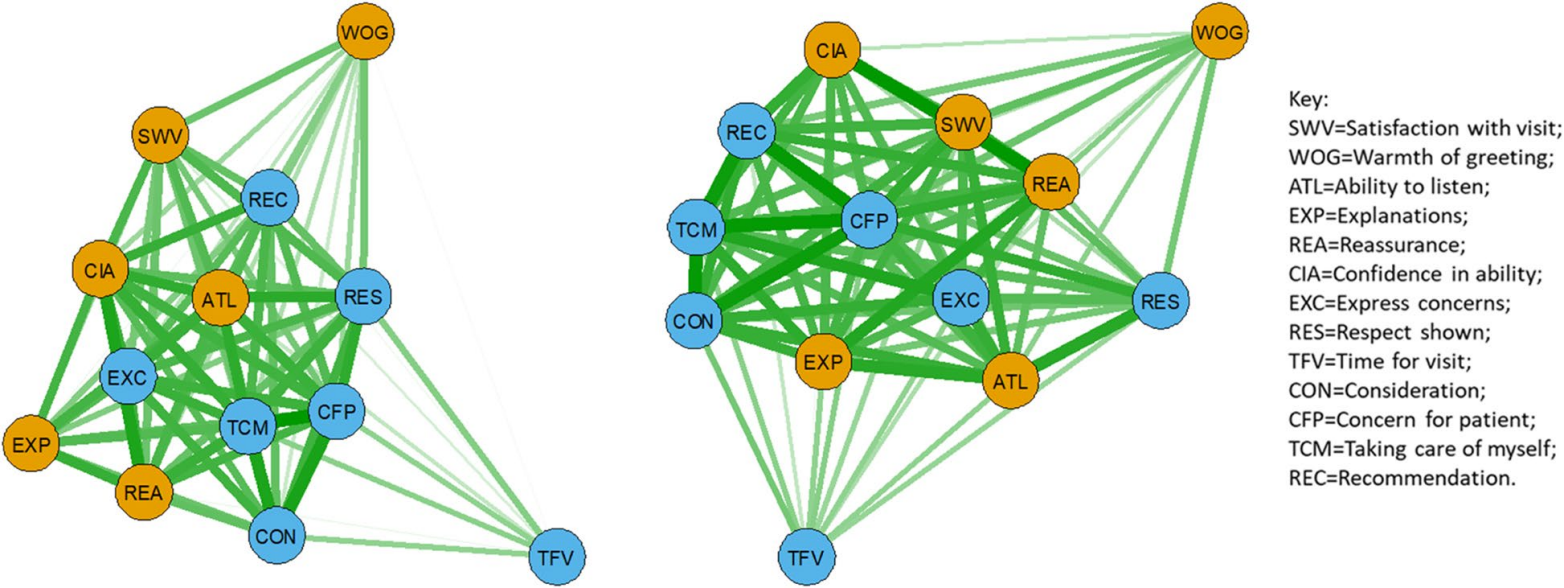
Psychometric network analysis showing mean-score item associations for (left figure) PEP doctors (*n* = 221) and (right figure) AGPT doctors (*n* = 355), with thickness of line related to strength of association (inter-item correlations rescaled between 0 and 1). The nodes are grouped by principal component (brown = interpersonal communication, blue = caring, Table 3) and the layout is ‘spring’

When PEP and AGPT doctors were separated, ‘Ability to listen’ was central in the PEP network with strong links to ‘Expressing concern’ and ‘Taking care of myself’ (Fig. 4, left). For the AGPT network, ‘Concern for patient’ was central with strong links to ‘Taking care of myself’, ‘Consideration’ and ‘Recommendation’. Both structures reveal strong links between ‘Confidence in ability’ and ‘Satisfaction with visit’.

**Fig. 5:**
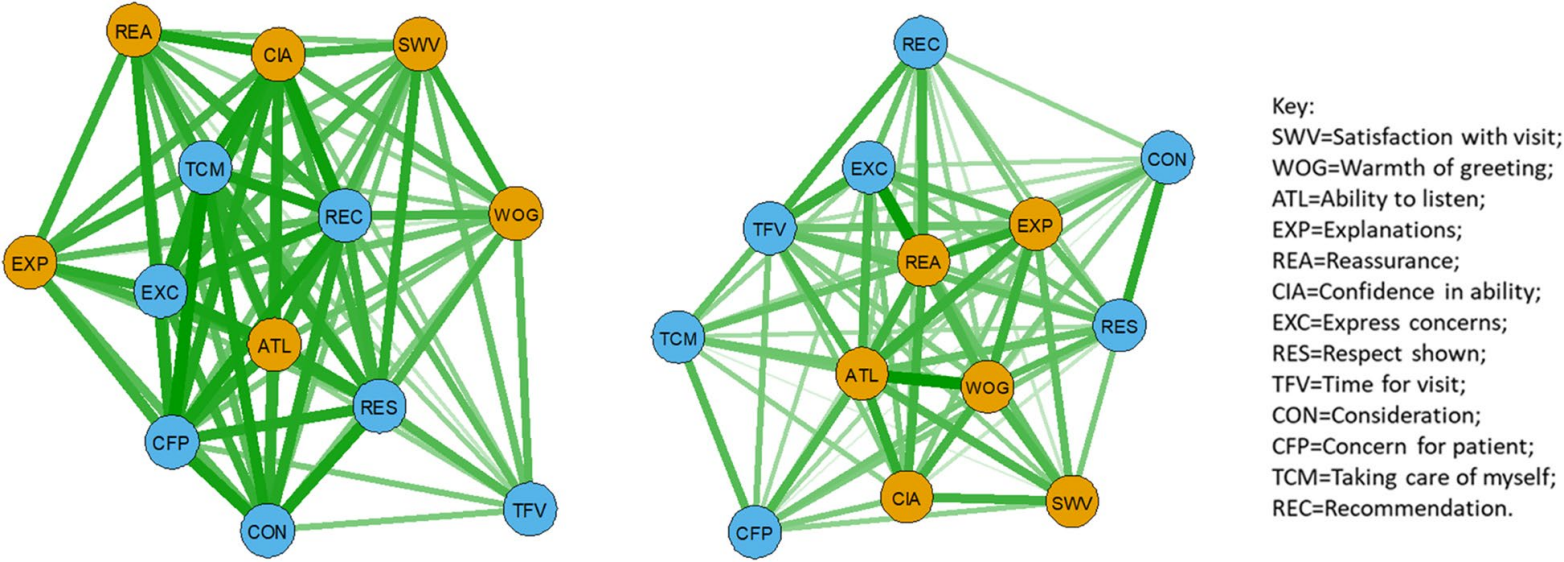
Psychometric network analysis comparing inter-item associations for PEP doctors in bottom tenth percentile (left, *n* = 22) with PEP doctors in top tenth percentile (right, *n* = 22), with thickness of line related to strength of association (inter-item correlations rescaled between 0 and 1). The nodes are grouped by principal component (brown = interpersonal communication, blue = caring, Table 3) and the layout is ‘spring’

When PEP doctors with scores within the bottom 10th percentile (≤82.19%) were compared with PEP doctors with scores in the top 10th percentile (≥96.2%), caring/empathy items were located more centrally for the former group (Fig. 5, left) while interpersonal skills were more central for the latter (Fig. 5, right). In particular, ‘Reassurance’, ‘Ability to listen’, ‘Warmth of greeting’ and ‘Explanations’ formed a tight central cluster for the top PEP doctors.

**Fig. 6:**
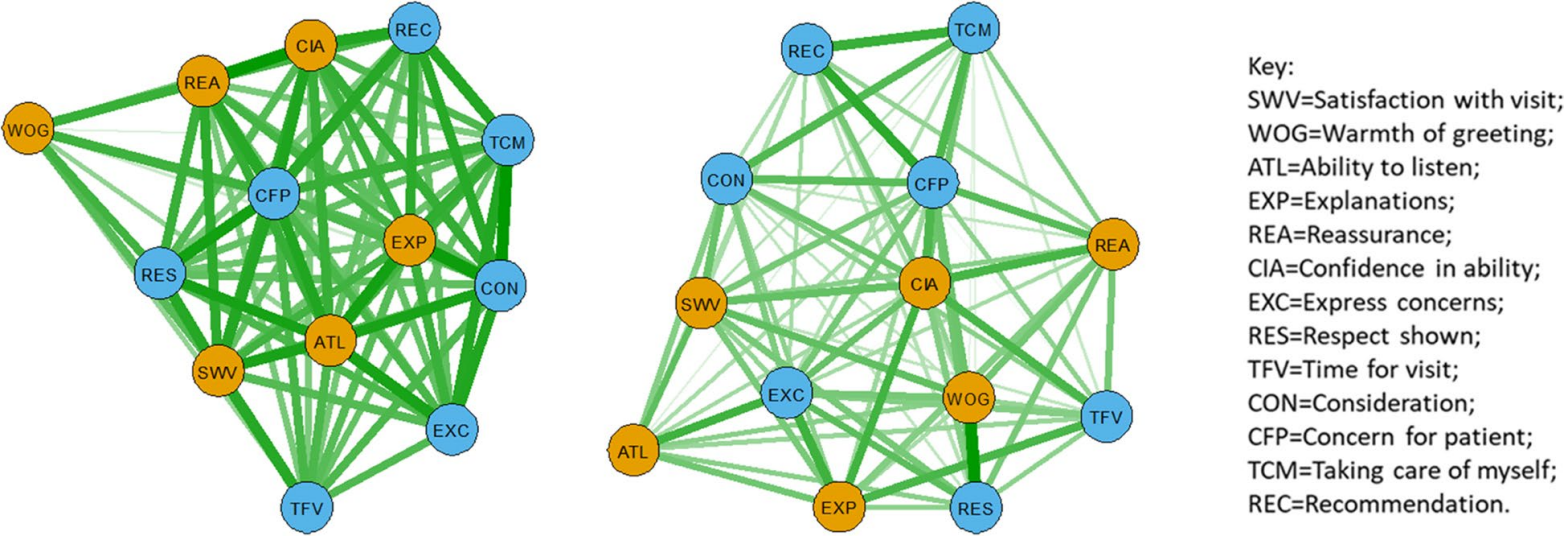
Psychometric network analysis comparing inter-item associations for AGPT doctors in bottom tenth percentile (left, *n* = 35) with AGPT doctors in top tenth percentile (right, *n* = 35), with thickness of line related to strength of association (inter-item correlations rescaled between 0 and 1). The nodes are grouped by principal component (brown = interpersonal communication, blue = caring, Table 3) and the layout is ‘spring’

When AGPT doctors with scores in the bottom 10th percentile (≤85.15%) were compared with AGPT doctors with scores in the top 10th percentile (≥95.76%), interpersonal skills consisting of ‘Confidence in ability’, ‘Warmth of greeting’, ‘Reassurance’ and ‘Explanations’ formed a central core for the latter group (Fig. 6, right). ‘Ability to listen’, ‘Explanations’ and ‘Concern for patients’ formed a central core for the former group (Fig. 6, left).

**Fig. 7:**
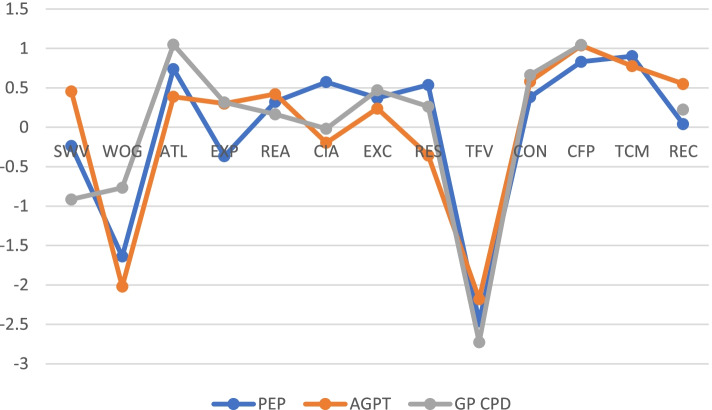
Strength of network nodes expressed in standardized summed item correlations for each group of doctors, with increasing values indicating increasing strength. Note that item TCM is missing for GP CPD doctors (see network figures for names of items)

Standardized strength values are shown in Fig. 7 for each of the doctor networks (GP CPD, PEP, AGPT) and indicate that ‘Ability to listen’ has strong connections in all three networks, followed by ‘Concern for patient’ and ‘Consideration’. Weakest nodes in terms of influence are ‘Warmth of greeting’ and ‘Time for visit’.

## Discussion and conclusion

The results presented here further our understanding of the communication skills and professionalism of doctors undertaking GP training, as perceived by their patients. This is also the first comparison of these skills between doctors on the AGPT and PEP pathways to RACGP Fellowship. With over 21,000 patient responses to 576 doctors undertaking GP training, and over 36,000 patient responses to over 900 GPs, the results presented here would appear to have validity in terms of margin for error and representativeness. This is also partially supported by consistency analysis of the data, which shows good agreement among patients about how to interpret the questionnaire, as well as the power analysis. Patient response rates for questionnaires completed through convenience sampling on site vary from 72 to 78%. These compare favourably with postal response rates (typically 20 to 60%) and are in line with previous patient satisfaction studies [26] as well as considered ‘high’ to ‘very high’ in the context of minimizing potential for non-response bias [27].

One of the impacts of ‘big data’ is that small differences between groups tend to be identified as significant because of the large numbers involved. Differences that appear minor with limited sample sizes can become statistically significant as the quantity of data grows, enabling finer significant discriminations to be made [20]. For instance, the difference between an average GP CPD score and an average PEP/AGPT score is only 0.8% (Table 2). The discussion below needs to be interpreted in the context of all three doctor groups achieving scores in the very good to excellent range (averages over 90%). Nevertheless, the small differences that are statistically significant may be useful for identifying trends that have functional significance for training programs, as identified below.

Patients were most satisfied with their experience with GP CPD doctors, followed by AGPT and PEP doctors (Table 4). In particular, patients had greatest confidence in the ability of GP CPD doctors. Patients were more satisfied with AGPT doctors than PEP doctors on ‘Explanations’, ‘Time for visit’, ‘Express concerns’ and ‘Ability to listen’. Percentile analysis showed patients rated AGPT doctors higher than PEP doctors until the 80th percentile (Fig. 2). Patients rated the very top PEP doctors (90th percentile) as better than the top AGPT doctors, with both still rated below GP CPD doctors. Patients rated AGPT doctors better than GP CPD doctors at the very lowest 10th percentile (Fig. 2, 85.15% vs 84.95). Female patients gave higher scores than male patients, and patients gave higher scores for visits to their usual doctor (Fig. 1). These aspects could have benefitted PEP doctors due to greater proportion of such patients in comparison to AGPT doctors (Table 2). PEP doctors are already working in General Practice on entry to their program, whilst AGPT registrars are placed into a practice on entry and so do not have an established patient load. Patients rated their doctors under two, equally balanced, previously identified components of interpersonal communication and caring/empathy (Table 3). These components appear to be consistent across all three doctor groups studied here.

Network analysis showed that all doctor groups had strong connections between ‘Concern for patient’ and ‘Consideration’ (Fig. 3). ‘Reassurance’ and ‘Confidence in ability’ were also strongly linked, based on patient feedback, for PEP and AGPT doctors (Fig. 3, left). For GP CPD doctors, strong links were demonstrated between ‘Ability to listen’, ‘Explanations’ and ‘Reassurance’ (Fig. 3, right). ‘Ability to listen’ was also linked strongly with ‘Concern for patient’, ‘Consideration’, ‘Reassurance’ and ‘Express concerns’. When separate networks for PEP and AGPT doctors were compared (Fig. 4), ‘Respect’ was central for PEP doctors, with strong links to ‘Concern for patient’, ‘Take care of myself’ and ‘Confidence in ability’. For APGT doctors, ‘Concern for patient’ was central, with strong links to ‘Take care of myself’, ‘Consideration’ and ‘Recommendation’.

The lowest scoring PEP doctors were distinguished from the top scoring PEP doctors by the centrality of care/empathy items for the former group and interpersonal communication skills for the latter group (Fig. 5). This pattern was repeated to some extent for AGPT doctors (Fig. 6). GP CPD doctors were identified by ‘Ability to listen’ being central and strongly related to other items (Fig. 3, right). Future studies could usefully study the relationship between empathy and caring on the one hand, and communication and interpersonal skills on the other, to identify ways in which practitioners may be able to better communicate that they care so that patients gain more confidence in the diagnosis and advice provided. For instance, methods involving scheduled follow-up discussions either via email or in person, or requesting feedback from the patient on how the management regime is progressing, could be possible ways to demonstrate empathy and care through further communication. Studies focused on these particular aspects of care, confidence and communication could lead to the gap between the lowest scoring doctors and the highest scoring doctors reducing even further.

One implication of these results is that the perception that IMGs and OTDs may lack the ‘soft skills’ to successfully practice in their new country will need revising. Our results show that IMGs and OTDs perform similarly with respect to communication and professionalism skills as their locally trained counterparts. However, our network analysis indicates that there may be issues of ‘connectedness’ and difference in priority between such skills that may need further exploration. In particular, the relationship between interpersonal communication and caring/empathy dimensions can vary according to background and training. This is consistent with the literature indicating that IMGs and OTDs can have difficulty adjusting to new cultures, communication styles, languages (including slang), health systems and health beliefs [6, 12, 28, 29]. A practical suggestion may be for training programs to be enhanced to integrate interpersonal skills and caring/empathy skills more fully, with the GP CPD network being used as a benchmark, to complement recent and similar recommendations for changes in medical undergraduate courses [30]. There is also growing interest in the use of feedback for debriefing and development purposes [31]. For instance, anonymous patient-doctor sessions could be recorded and PEP/AGPT doctors then asked to rate the patient’s experience using the patient questionnaire, with comparisons made against real patient data (all subject to ethical approval and permission of all parties concerned). Network analysis indicates that focusing on ability to listen and concern for patient (the two most central items) might be useful for enhancing the ability of PEP and AGPT doctors to appreciate the importance of communication and care/empathy for patient-centredness, which will likely benefit their registration pathway process. Given that the PEP program is oriented towards self-directed education with variable supervisory arrangements, as well as located predominantly outside major cities, a challenge for future program development may be to identify methods for enhancing mechanisms, such as greater contact with experienced GPs, for helping trainee GPs to enhance their communication and interpersonal skills as identified above.

Interpersonal communication is now accepted as a fundamental clinical skill in medical practice [32, 33], with good communication establishing trust between patient and doctor as well as leading to better exchange of information. Listening, explaining and empathizing can have a major effect on patient health status and satisfaction. The psychometric network for experienced GPs (Fig. 3, right), for instance, shows a tight and central clustering of interpersonal communication component items (‘Ability to listen’, ‘Explanation provided’) with empathy component items (‘Expressing concerns’, ‘Consideration’, ‘Concern for patient’). Experienced GPs also receive the highest satisfaction ratings. Networks for PEP and AGPT doctors show different relationships between items, leading to speculative hypotheses and interpretations concerning differences between doctor training groups in terms of possible clinical performance in comparison with experienced GPs. However, in the absence of other sources of data concerning clinical effectiveness of consultation, these networks only identify possible areas for changes in training programs and additional support for doctor groups, as discussed above. No conclusions can be drawn from these networks concerning the clinical effectiveness of consultations by any doctor or doctor group, or how these differences affect clinical treatment of patients and patient satisfaction.

The aim of the research was to understand how doctors undertaking the PEP and AGPT pathways to GP Fellowship, and Fellowed GPs compare regarding communication skills and professionalism. This research demonstrates the high quality of patient care given by PEP and AGPT doctors, as well as Fellowed GPs, and highlights the interrelationships between professional skills, including which skills are focal or central to each doctor group. Overall, each group of doctors has excellent performance, and doctors on GP Fellowship pathways can aspire to consolidate their skills cohesively to further improve their performance, as seen with the experienced GPs. Given that the PEP program is oriented towards self-directed education with variable supervisory arrangements in geographically diverse practice locations, a challenge for future program development may be to identify methods for strengthening mechanisms, such as greater contact with experienced GPs, to help trainee GPs enhance their communication and interpersonal skills as identified above. This recommendation is in line with colleague feedback obtained for the same group of trainees, which showed that colleagues, while rating the clinical skills of PEP trainees highly, identified a gap in communication skills in comparison with AGPT GPiT [34].

## Limitations

Limitations of this study include the variable numbers of doctors used for each part of the analysis due to data being collected at different times for such a large-scale study. The later stage of data collection (i.e., early to mid-2020) was affected by COVID-19, leading to early termination of data collection. While this study reports on the quantitative aspects of the study, further work involving observations and qualitative analysis, including qualitative analysis of comments supplied by patients, is required to identify specific behavioural patterns of doctors that may affect ratings provided. Additionally, there is limited demographic data available for PEP doctors, and due to the eligibility criteria it has been assumed that the majority of doctors undertaking this pathway to RACGP Fellowship are IMGs and OTDs. While some aspects of patient demographics were taken into account in the analysis, no sociodemographic or ethnic aspects of individual doctors being rated were collected to ensure lack of personal identification. There may be bias against doctors based on sociodemographic and ethnic factors, although the small difference in average ratings between the two doctor groups would suggest that differences between patient groups were larger than differences between doctor groups. The possible effects of such bias on ratings are not measured in this study. Finally, while response rates through convenience sampling are high to very high, there is unknown potential bias in non-responses which can limit the generalizability of the results to other patient populations, such as questionnaires only being completed if patients were satisfied with their visit or, conversely, patients more likely to complete their questionnaire because they were unhappy with their visit. An assumption made in this study is that any patient bias is randomly distributed and contributes equally to all doctor ratings.

## Supplementary Information

**Additional file 1.** Questionnaire Items (Long and Short Versions).

## Data Availability

The datasets used during the current study are available from the corresponding author on reasonable request from an institutional address. Interested readers are also asked to contact the corresponding author for more details concerning the full content of the questionnaire and its layout.

## Abbreviations

IMG: International medical graduate
OTD: Overseas trained doctor
MSF: Multisource feedback
RACGP: Royal Australian College of General Practitioners
PEP: Practice Experience Program
AGPT: Australian General Practice Training Program
GPiT: General practitioner in Training
GP: General practitioner
CPD: Continuing professional development
TIDieR: Tgemplate for intervention descriptoin and replication
SPSS: Statistical Package for Social Sciences (IBM software)
CFEP: Client Focused Evaluation Program (company based in Brisbane)
ANOVA: Analysis of variance
PCA: Principal component analysis
KMO: Kaiser-Meyer-Olkin test
ICC: Intraclass correlation coefficient
SNR: Signal-to-noise ratio
