# Supplementary material for: A comparison of patient appraisal of professional skills for GPs in training participating in differing education programs
Source: BMC Med Educ. 2022 Sep 10;22:669. doi: 10.1186/s12909-022-03733-9 (PMC9462893; doi:10.1186/s12909-022-03733-9)
Supplement: Supplementary file 1 — Additional file 1. Questionnaire Items (Long and Short Versions). [file 12909_2022_3733_MOESM1_ESM.docx]

**Additional file 1: Questionnaire Items (Long and Short Versions)**

The patient questionnaire consists of 13 Likert scale items, each with five labels (‘poor’, ‘fair’, ‘good’, ‘very good’, ‘excellent’), with patients asked to indicate which one of the labels most appropriately describes their experience of the consultation they have just had with the doctor.

The full wording of the items and the short versions used when presenting results are as follows:

| ***Item*** | ***Long form on questionnaire*** | ***Short form in this report*** |
| --- | --- | --- |
| Q1 | My overall satisfaction with this visit to the doctor is | Satisfaction with visit |
| Q2 | The warmth of the doctor's greeting to me was | Warmth of greeting |
| Q3 | On this visit I would rate the doctor's ability to really listen to me as | Ability to listen |
| Q4 | The doctor's explanations of things to me were | Explanations |
| Q5 | The extent to which I felt reassured by this doctor was | Reassurance |
| Q6 | My confidence in this doctor's ability is | Confidence in ability |
| Q7 | The opportunity the doctor gave me to express my concerns or fears was | Express concerns |
| Q8 | The respect shown to me by this doctor was | Respect shown |
| Q9 | The amount of time given to me for this visit was | Time for visit |
| Q10 | This doctor's consideration of my personal situation in deciding a treatment or advising me was | Consideration |
| Q11 | This doctor's concern for me as a person on this visit was | Concern for patient |
| Q12 | The extent to which the doctor helped me to take care of myself was | Take care of myself |
| Q13 | The recommendation I would give to my friends about this doctor would be | Recommendation |
